# Non-contrast computed tomography-based radiomics for staging of connective tissue disease-associated interstitial lung disease

**DOI:** 10.3389/fimmu.2023.1213008

**Published:** 2023-10-06

**Authors:** Songnan Qin, Bingxuan Jiao, Bing Kang, Haiou Li, Hongwu Liu, Congshan Ji, Shifeng Yang, Hongtao Yuan, Ximing Wang

**Affiliations:** ^1^ Department of Radiology, Shandong Provincial Hospital, Shandong University, Jinan, Shandong, China; ^2^ Department of Radiology, Shandong Provincial Hospital Affiliated to Shandong First Medical University, Jinan, Shandong, China; ^3^ Department of Radiology, Qilu Hospital, Cheeloo College of Medicine, Shandong University, Jinan, Shandong, China

**Keywords:** connective tissue diseases, interstitial lung diseases, radiomics, machine learning, computed tomography

## Abstract

**Rationale and introduction:**

It is of significance to assess the severity and predict the mortality of patients with connective tissue disease-associated interstitial lung disease (CTD-ILD). In this double-center retrospective study, we developed and validated a radiomics nomogram for clinical management by using the ILD-GAP (gender, age, and pulmonary physiology) index system.

**Materials and methods:**

Patients with CTD-ILD were staged using the ILD-GAP index system. A clinical factor model was built by demographics and CT features, and a radiomics signature was developed using radiomics features extracted from CT images. Combined with the radiomics signature and independent clinical factors, a radiomics nomogram was constructed and evaluated by the area under the curve (AUC) from receiver operating characteristic (ROC) analyses. The models were externally validated in dataset 2 to evaluate the model generalization ability using ROC analysis.

**Results:**

A total of 245 patients from two clinical centers (dataset 1, n = 202; dataset 2, n = 43) were screened. Pack-years of smoking, traction bronchiectasis, and nine radiomics features were used to build the radiomics nomogram, which showed favorable calibration and discrimination in the training cohort {AUC, 0.887 [95% confidence interval (CI): 0.827–0.940]}, the internal validation cohort [AUC, 0.885 (95% CI: 0.816–0.922)], and the external validation cohort [AUC, 0.85 (95% CI: 0.720–0.919)]. Decision curve analysis demonstrated that the nomogram outperformed the clinical factor model and radiomics signature in terms of clinical usefulness.

**Conclusion:**

The CT-based radiomics nomogram showed favorable efficacy in predicting individual ILD-GAP stages.

## Highlights

- Assessment of the severity of CTD-ILD is difficult by conventional imaging modalities.
- Radiomics nomogram can predict the GAP stage with improved efficacy in comparison to clinical factors.
- The CT-based radiomics nomogram might provide treatment guidance for CTD-ILD.

## Introduction

Interstitial lung diseases (ILDs) are spread parenchymal lung disturbances frequently associated with connective tissue disease (CTD) (1). All patients with CTD face the risk of ILD, which may occur at any point during the period of CTD, even the first clinically apparent manifestation of their CTD (2). ILDs are mostly seen in systemic sclerosis (SSc), rheumatoid arthritis (RA), Sjögren’s syndrome (SjS), systemic lupus erythematosus (SLE), idiopathic inflammatory myositis [including polymyositis (PM)/dermatomyositis (DM) and anti-synthetase syndrome], and mixed connective tissue disease (MCTD) (3).

On account of the shortage of randomized controlled trials and recommendations, identifying which treatment to implement for CTD-ILD is currently a predicament for clinicians (3–5). Although it has been reported that ILD is associated with early mortality, which is responsible for up to 35% of CTD-related deaths in some cohorts (6–10), rushing into medical intervention may result in unnecessary drug toxicant exposure on stable patients and opportunity of infection (11, 12). Thus, staging approaches across CTD-ILD for individual treatment need to be developed to relieve impairments (3, 9).

The GAP (gender, age, and pulmonary physiology) index and staging scale were proposed for predicting the mortality risk of idiopathic pulmonary fibrosis (IPF) patients by Ley et al. (13) in 2012 and subsequently improved and validated to adapt non-IPF ILDs by Ryerson et al. (14). The ILD-GAP index scale used gender, age, predicted forced vital capacity (FVC), and diffusion capacity of carbon monoxide (DLCO) to estimate the severity and predict the mortality in patients with chronic ILD. It has been validated to be accurate in various kinds of CTDs (15–19).

Computed tomography (CT) scan remains the main method for ILD diagnosis at present because it is a noninvasive sensitive technique for detecting lung involvement in CTD patients (20–23). CT imaging together with PFT is the gold standard to assess and stage the severity of ILD noninvasively at present (24). However, visual analysis of ILDs on CT image presents difficulty in providing prognosis information because different stages of ILD share overlapping imaging features, conferring difficulty in diagnosing and assessing the severity of ILD by conventional imaging modalities. Radiomics technology can extract a large number of high-dimensional features from CT images, which could make up for the shortcomings of visual assessment. Radiomics has been investigated for diagnosis and prognosis in many diseases, but mostly in different kinds of tumors (25, 26). Radiomics were able to predict mortality and response to treatment in patients with CTD-ILDs, exploring prognostic information hiding beneath CT images that visual assessment has difficulty in acquiring (27, 28). There were correlations between radiomics features and GAP stages, indicating potentials in radiomics to stage patients in CTD-ILDs (29). In the present study, we aimed to establish a CT-based radiomics nomogram to differentiate and stage CTD-ILD phases.

## Materials and methods

### Patients

Authorization of the institutional review board was granted, and informed consent was waived.

Patients who were clinically diagnosed with CTD (SSc, RA, SjS, PM/DM, SLE, and MCTD) from June 2015 to June 2021 in Shandong Provincial Hospital Affiliated to Shandong First Medical University (dataset 1) and Qilu Hospital of Shandong University (dataset 2) were screened consecutively. Patients were included when they satisfied all of the following conditions: 1) diagnosed with CTD fulfilling the American college of rheumatology/European league against rheumatism (ACR/EULAR) or other acknowledged classification criteria (30–35), 2) underwent CT scan with signs of ILD within 3 months after clinical diagnosis, and 3) underwent pulmonary function tests (PFTs) and laboratory examination within 30 days before or after the CT scan. Patients were ruled out when they fulfilled any of the following conditions: 1) diagnosed with tumors in the lung; 2) diagnosed with idiopathic interstitial pneumonia, sarcoidosis, or any disease other than CTD that may lead to ILD; 3) any surgical history of the thorax; and 4) incomplete demographic or clinical data. The PFT indices included the percentage predicted values (%predicted) of forced expiratory volume in 1 s (FEV1), FVC, total lung capacity (TLC), and diffusion capacity of carbon monoxide. The ILD-GAP index was calculated according to Ryerson et al. (14). The patients were divided into two groups where Group I included patients with ILD-GAP index ≤1, and Group II included patients with ILD-GAP index >1. All patients were followed up until October 2022 and all-cause mortality was the endpoint. The predictive performance of the ILD-GAP index was evaluated by using univariate variable Cox regression and Harrell’s C index. Patients in dataset 1 were then randomly split into training and internal validation cohorts at a ratio of 7:3. The external validation cohort was composed of patients in dataset 2.

### CT image acquisition and evaluation

All CT examinations were performed in supine position with maximum inspiration. The detailed scanning parameters are shown in **Supplementary Table S1**.

The CT images were reviewed by two radiologists (Qin S.N. with 5 years and Wang X.M. with 20 years of thoracic imaging experience) without awareness of any other characteristics of the patients, and divergences were unified by consensus. The presence of visual characteristics of ILD (yes/no), including subpleural lines, reticular changes, honeycombing, pulmonary emphysema, and traction bronchiectasis, was evaluated case-by-case. All CT characteristics mentioned met the Fleischner Society criteria proposed in 2008 (36). The proportion (%) of the parenchymal extent in total lung volume was calculated using the pneumonia diagnosis module of Dr. Turing^®^ artificial intelligence-assisted diagnosis system (Huiying Medical Technology Co., Ltd.).

### Three-dimensional lung segmentation and extraction of texture features

All CT images were reprocessed by resampling into 1.0-mm-thick slices and intensity normalization into a range of [–1, 1]. The region of interest (ROI) segmentation within the borders of the right lung (window width = 1,500; window level = -750) was manually delineated using the 3D Slicer software (version 4.11, www.slicer.org). The outline of the ROI was contoured avoiding the hilar vessels. The left lung was not segmented, since the presence of the heart may add to the difficulties of segmentation and potentially lead to alterations in the results.

Extraction of the radiomics features was conducted through the Radcloud platform (www.huiyihuiying.com, Huiying Medical Technology Co., Ltd.). Compliant with the definitions of the Imaging Biomarker Standardization Initiative (37), 1,409 radiomics features altogether were retrieved from each ROI, whose information are in the **Supplementary Results**.

Interclass and intraclass correlation coefficients (ICCs) were applied through the following steps: 20 cases containing 10 Group I patients and 10 Group II patients were randomly selected to perform ROI segmentation by the readers. Reader 1 repeated the segmentation a month later. Segmentation was considered well matched in terms of the interobserver reliability and intraobserver reproducibility when the ICC value was greater than 0.75. Reader 1 then completed the rest of the segmentation procedures.

### Construction of the clinical model

The clinical factor model comprised significant difference variables between the two groups (p< 0.05) selected by univariate logistic regression analysis, including clinical data, laboratory examinations, and visual CT characteristics. Gender, age, and PFT parameters were excluded to prevent data leakage of the models. Then, the model was built using multivariable logistic regression analysis. Odds ratios (ORs) with 95% confidence intervals (CIs) were calculated for significantly correlated variables.

### Construction of the radiomics model and the combined model

To prevent model overfitting, dimensionality reduction of the radiomics features was performed before the signature to be constructed. In the training cohort, the features for constructing the radiomics model should satisfy the following conditions: interobserver and intraobserver ICCs exceeding 0.75; remarkable variant from one another as confirmed by analysis of variance; and selected as major contributories for predicting by bringing into the least absolute shrinkage and selection operator (LASSO) regression model. Finally, the radiomics model was constructed using the support vector machine (SVM) with selected features. The radiomics score (Rad-score) representing the weighting coefficient of the features for each patient was calculated.

Incorporating the significant clinical factors and the radiomics signature, a radiomics nomogram was constructed using multivariable logistic regression analysis. Variance inflation factors (VIFs) of the predictors were calculated for multicollinearity. A calibration curve was drawn to estimate the calibration of the combined model. The goodness of fit of combined model was estimated using the Hosmer–Lemeshow test.

### Evaluation of model capabilities

The classification performance of the clinical factor model, radiomics model, and combined model to differentiate Group II CTD-ILD from Group I was represented by the area under the curve (AUC) from receiver operating characteristic (ROC) curves. The comparison between the three models was assessed using the likelihood ratio test (LRT). The net benefits for a range of threshold probabilities were calculated by applying decision curve analysis (DCA) to measure the clinical benefit of the combined model. All three models were externally validated based on dataset 2 to evaluate the model generalization ability using ROC analysis.

### Statistical analysis

SPSS (version 26.0) and R software (version 3.5.1) were used to perform statistical tests and analyses. Significantly different clinical characteristics were detected using chi-square test, Fisher exact test, or Mann–Whitney U test, where appropriate. The DeLong test was conducted to test whether the AUC of the models based on the same cohort significantly varied. Categorical and continuous variables are shown by form of frequency (percentages), mean ± standard deviation, or median (interquartile range), where appropriate. The regression analysis, nomogram development, calibration plots, ROC analysis, and DCA were performed by the packages “rms,” “glmnet,” “pROC,” and “dcurves” in R. A two-tailed p value of<0.05 was regarded as indicating significant variation.

## Results

### Patient characteristics and the clinical model

**Figure 1:**
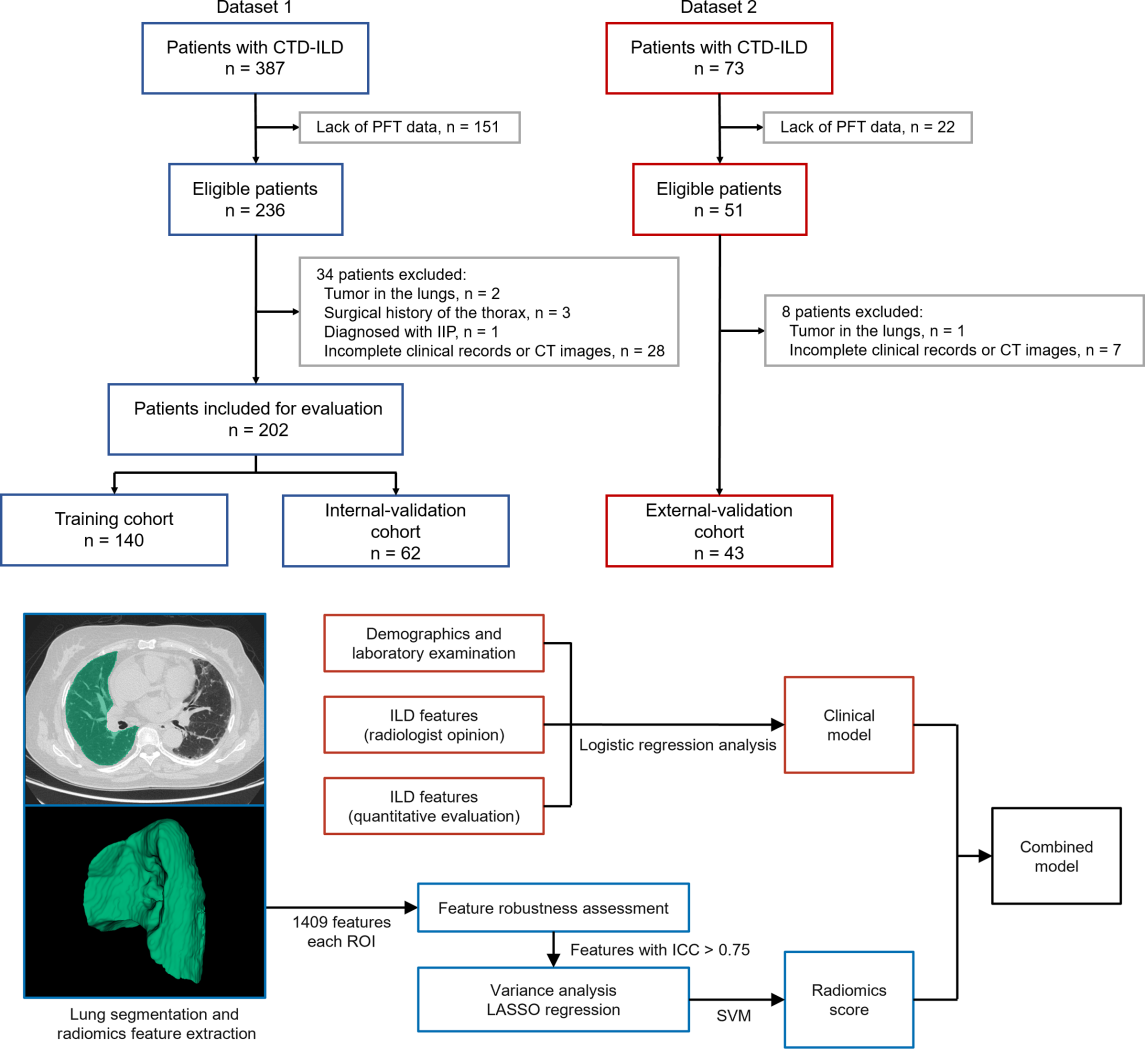
Flowchart of the study patients.

**Figure 1** showed the process of patients’ enrollment and model construction. Eventually, a total of 245 patients (dataset 1, n = 202; dataset 2, n = 43) were enrolled in this study. Group I included 158 ILD-GAP stage I patients, while Group II included 40 ILD-GAP stage II and four stage III patients. The distribution of their CTD subtypes is detailed in **Supplementary Table S2**. **Table 1** listed the baseline patient characteristics in dataset 1. The ILD-GAP index exhibited increasing mortality in patents with higher stages by univariate Cox regression (Hazard Ratio, 5.364; 95% CI, 1.994–14.424; p = 0.01) and showed acceptable mortality predictive performance (C-index 0.703) in some of the patients of dataset 1 (n = 74). More detailed follow-up information was shown in the **Supplementary Material**. **Table 2** exhibited the outputs of univariable and multivariable logistic regression analyses, which suggested that pack-years of smoking and traction bronchiectasis remained as independent predictors. Patients with a larger number of cigarettes smoked (OR, 1.036; 95% CI, 1.010–1.063) or traction bronchiectasis on CT image (OR, 3.705; 95% CI, 1.222–11.239) tended to have a higher mortality. We examined the two predictors in dataset 2, which showed parallel results (**Supplementary Table S4**).

**Table 1:** Patients’ baseline clinical factors between group I with GAP stage I patients and group II with GAP stage II/III patients in dataset 1.

Variables	Training cohort (n=140)			Internal-validation cohort (n=62)		
	Group I	Group II	*p* value	Group I	Group II	*p* value
Demographics				1.000	0.000	
Number (n)	110	30		48	14	
Gender			<0.001			0.430
Male	22 (20%)	17 (56.7%)		15 (31.3%)	2 (14.3%)	
Female	88 (80%)	13 (43.3%)		33 (68.8%)	12 (85.7%)	
Median age (range), years	52.5 (18-79)	66 (38-81)	<0.001	50 (20-73)	64.5 (50-76)	0.022
BMI, kg/m^2^*	24.1 ± 3.7	24.6 ± 4.3	0.381	23.3 ± 3.7	22.7 ± 3.7	0.490
Pack-years of smoking	0.00 [0.00-0.00]	0.00 [0.00-17.50]	0.002	0.00 [0.00-0.00]	0.00 [0.00-0.75]	0.022
Pulmonary arterial hypertension**	12 (10.9%)	3 (10%)	0.631	1 (2.08%)	2 (14.2%)	1.000
Symptoms						
Fever (y/n)	22 (20%)	4 (13.3%)	0.405	12 (25%)	1 (7.14%)	0.284
Cough (y/n)	46 (41.8%)	10 (33.3%)	0.400	19 (39.6%)	8 (57.1%)	0.244
Cutaneous (y/n)	33 (30%)	10 (33.3%)	0.726	13 (27.1%)	2 (14.2%)	0.529
Joint tenderness and swelling (y/n)	67 (60.9%)	14 (46.7%)	0.161	29 (60.4%)	10 (71.4%)	0.453
Chest distress and dyspnea (y/n)	49 (44.5%)	15 (50%)	0.595	14 (29.2%)	10 (71.4%)	0.004
Pulmonary function						
FVC% predicted*	85.2 ± 19.2	66.4 ± 15.6	<0.001	86.0 ± 17.5	58.0 ± 16.2	<0.001
FEV_1_% predicted*	86.9 ± 18.0	70.0 ± 16.7	<0.001	87.2 ± 18.4	61.5 ± 22.3	<0.001
TLC% predicted*	84.4 ± 16.0	51.2 ± 26.0	<0.001	84.0 ± 16.4	55.9 ± 14.7	<0.001
DL_CO_% predicted*	63.8 ± 14.4	28.9 ± 16.8	<0.001	61.3 ± 15.9	32.9 ± 15.0	<0.001
Laboratory Examinations						
ESR	26.00 [14.00-58.00]	29.00 [13.25-58.00]	0.984	29.50 [20.00-73.00]	49.50 [25.50-75.25]	0.429
CRP	3.38 [1.09-15.16]	12.41 [1.27-29.65]	0.143	4.74 [1.81-32.74]	5.76 [2.70-12.62]	0.556
PCT	0.01 [0.01-0.01]	0.01 [0.01-0.02]	0.771	0.01 [0.01-0.03]	0.01 [0.01-0.04]	0.417
ASO	4 (3.6%)	1 (3.33%)	1.000	5 (10.4%)	0 (0%)	0.579
RF	44 (40%)	13 (43.3%)	0.742	21 (43.8%)	8 (57.1%)	0.377
CCP	24 (21.8%)	10 (33.3%)	0.192	10 (20.8%)	4 (28.6%)	0.806
APLA	27 (24.5%)	4 (13.3%)	0.190	13 (27.1%)	3 (21.4%)	0.938
ANA	102 (92.7%)	28 (93.3%)	1.000	41 (85.4%)	10 (71.4%)	0.419
ANCA	5 (4.5%)	2 (6.7%)	1.000	3 (6.3%)	1 (7.1%)	1.000
Features of ILD on HRCT						
Subpleural lines (y/n)	101 (91.8%)	28 (93.3%)	1.000	43 (89.6%)	13 (92.9%)	1.000
Reticular changes (y/n)	92 (83.6%)	27 (90%)	0.564	40 (83.3%)	11 (78.6%)	0.990
Honeycombing (y/n)	37 (33.6%)	13 (43.3%)	0.326	18 (37.5%)	11 (78.6%)	0.007
Pulmonary emphysema (y/n)	41 (37.3%)	16 (53.3%)	0.112	20 (41.7%)	11 (78.6%)	0.015
Traction bronchiectasis (y/n)	9 (8.2%)	7 (23.3%)	0.047	5 (10.4%)	6 (42.9%)	0.016
Proportion of parenchymal extent (%)	5.96 [2.22-13.75]	16.47 [4.67-22.65]	0.045	7.41 [2.57-13.94]	13.93 [7.09-27.47]	0.046

Categorical variables are presented as n (%). Continuous variables are listed as median (inter-quartile range, IQR) or *as mean ± standard deviation.

n number of patients, y/n yes/no, ESR erythrocyte sedimentation rate, PCT procalcitonin, ASO anti-streptolysin O, RF rheumatoid factor, CRP C-reactive protein, APLA anti-phospholipid antibodies, ANA antinuclear antibodies, ANCA antineutrophil cytoplasmic antibodies, CCP anti-cyclic citrullinated peptide antibodies, ILD interstitial lung disease.

**Expert opinion by echocardiography.

**Table 2:** Risk factors for Group II CTD-ILD in the training cohort.

Variables	Univariable analysis		Multivariable analysis	
	Odds ratio (95% CI)	p value	Odds ratio (95% CI)	p value
Gender	5.24 (2.21-12.35)	<0.001		
Age	1.08 (1.04-1.13)	<0.001		
BMI	1.04 (0.94-1.15)	0.475		
Pack-years of smoking	1.04 (1.01-1.06)	0.010	1.04 (1.01-1.06)	0.007
Pulmonary arterial hypertension	1.93 (0.45-8.20)	0.375		
Fever	0.62 (0.19-1.95)	0.409		
Cough	0.70 (0.30-1.63)	0.402		
Cutaneous	1.17 (0.49-2.76)	0.726		
Joint tenderness and swelling	0.56 (0.25-1.27)	0.164		
Chest distress and dyspnea	1.25 (0.56-2.79)	0.595		
FVC% predicted	0.94 (0.91-0.96)	<0.001		
FEV1% predicted	0.94 (0.92-0.97)	<0.001		
TLC% predicted	0.87 (0.82-0.92)	<0.001		
DLCO% predicted	0.86 (0.80-0.91)	<0.001		
ESR	1.00 (0.99-1.01)	0.937		
CRP	1.01 (1.00-1.02)	0.114		
PCT	0.48 (0.13-18.29)	0.694		
ASO	0.91 (0.10-8.49)	0.937		
RF	1.15 (0.51-2.60)	0.742		
CCP	1.79 (0.74-4.34)	0.196		
APLA	0.47 (0.15-1.48)	0.197		
ANA	1.10 (0.22-5.47)	0.909		
ANCA	1.50 (0.28-8.15)	0.639		
Consolidation	1.10 (0.22-5.47)	0.909		
Subpleural lines	1.25 (0.26-6.11)	0.785		
Reticular changes	1.76 (0.48-6.43)	0.392		
Honeycombing	1.51 (0.66-3.44)	0.328		
Pulmonary emphysema	1.92 (0.85-4.34)	0.116		
Traction bronchiectasis	3.42 (1.15-10.12)	0.027	3.71 (1.22-11.24)	0.021
Proportion of parenchymal extent	1.04 (1.01-1.07)	0.021	1.03 (1.00-1.07)	0.053

### Development of the radiomics model

**Figure 2:**
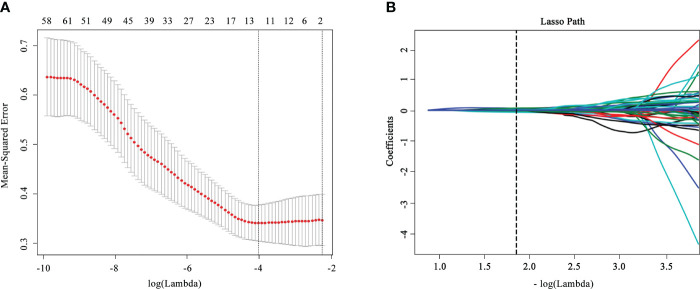
Feature selection and dimensionality reduction workflow. **(A)** Confirmation of the tuning parameter (λ) in the least absolute shrinkage and selection operator model. An optimal λ value of 0.015 with (vertical dash line) was selected. **(B)** The feature coefficients varied according to log(λ).


A total of 1,409 radiomics features were obtained from the CT images; 1,367 of them were examined to be of promising interobserver and intraobserver accordance (intraclass correlation coefficient >0.75). Seventy significantly different (p< 0.05) radiomics features selected went through the LASSO logistic regression analysis to choose the optimally related features (**Figure 2**). Eventually, nine features were put into radiomics model construction. **Supplementary Table S5** listed elaborated information of the features. The Rad-score was calculated according to the following equation:

$$\begin{array}{l} \text{Rad-score}=0.052\times \text{original\_shape\_Flatness}+0.0158\times \text{wavelet-HHL\_firstorder\_Kurtosis}+0.0381\\ \times \text{wavelet-HLH\_glcm\_SumSquares}+0.0422\times \text{wavelet-LHH\_firstorder\_Kurtosis}+0.0244\\ \times \text{wavelet-LHH\_glcm\_Autocorrelation}-0.0167\times \text{wavelet-LHL\_glrlm\_GrayLevelVariance}-0.0037\\ \times \text{wavelet-LHL\_glrlm\_LowGrayLevelRunEmphasis}-0.0656\\ \times \text{wavelet-LHL\_glszm\_SizeZoneNonUniformityNormalized}+0.0271\\ \times \text{wavelet-LLL\_glszm\_SmallAreaEmphasis} \end{array}$$

**Figure 3:**
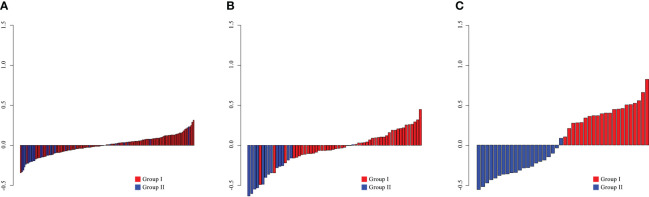
The radiomics scores for each patient in the training **(A)**, internal validation **(B)**, and external validation **(C)** cohorts.

The Rad-score was a tested statistically significant variant between the two groups (p< 0.05; **Supplementary Table S6**) and presented in **Figure 3**.

### Development of the combined model

**Figure 4:**
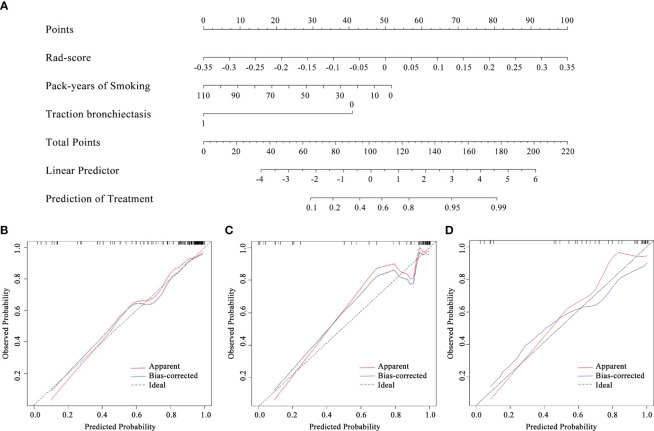
The radiomics nomogram **(A)** constructed combining pack-years of smoking, traction bronchiectasis, and Rad-score and the calibration curves of the radiomics nomogram in the training **(B)**, internal validation **(C)**, and external validation **(D)** cohorts.


By comprising the pack-years of smoking, traction bronchiectasis, and Rad-score, a combined model was built in the training cohort (**Figure 4A**). The VIFs of the predictors ranged from 1.04 to 1.08, indicating that there was no multicollinearity. The calibration curve of the radiomics nomogram is presented in **Figures 4B–D**, which represented acceptable calibration in the training cohort (p = 0.089), the internal validation cohort (p = 0.107), and the external validation cohort (p = 0.217) through the Hosmer–Lemeshow test. The nomogram score was calculated according to the following equation:

$$\begin{array}{c} \text{Nomogram score}=142.5744\;+\;142.8572\times \text{Rad-score}-0.4698\\ \times \text{pack-year of smoking}-40.8917\times bronchiectasis \end{array}$$

### The validation of the capabilities of the models

**Figure 5:**
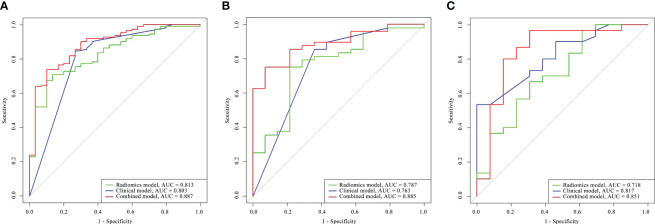
Comparison of the ROC curves for the radiomics model, clinical model, and combined model in the training cohort **(A)**, the internal validation cohort **(B)**, and the external validation cohort **(C)**.

The capability of the diagnostic efficiency for each model is presented in **Table 3**. The ROC curves of the clinical factor model and combined model are presented in **Figure 5**.

**Table 3:** Diagnostic performance of the clinical factor model, the radiomics signature, and the radiomics nomogram.

Model	Group	AUC (95% CI)	Sensitivity	Specificity	Accuracy	PPV	NPV	F1-score
Clinical factor model	Training cohort	0.803 (0.723–0.876)	0.891 (98/110)	0.633 (19/30)	0.836 (117/140)	0.899 (98/109)	0.613 (19/31)	0.895
	Internal validation cohort	0.763 (0.603–0.841)	0.875 (42/48)	0.571 (8/14)	0.806 (50/62)	0.875 (42/48)	0.571 (8/14)	0.875
	External validation cohort	0.817 (0.690–0.833)	0.533 (16/30)	1 (13/13)	0.674 (29/43)	1 (16/16)	0.481 (13/27)	0.696
Radiomics signature	Training cohort	0.813 (0.743–0.877)	0.736 (81/110)	0.7 (21/30)	0.729 (102/140)	0.9 (81/90)	0.42 (21/50)	0.81
	Internal validation cohort	0.787 (0.606–0.825)	0.667 (32/48)	0.786 (11/14)	0.694 (43/62)	0.914 (32/35)	0.407 (11/27)	0.771
	External validation cohort	0.718 (0.531–0.778)	0.633 (19/30)	0.692 (9/13)	0.651 (28/43)	0.826 (19/23)	0.45 (9/20)	0.717
Radiomics nomogram	Training cohort	0.887 (0.827–0.940)	0.818 (90/110)	0.8 (24/30)	0.814 (114/140)	0.938 (90/96)	0.545 (24/44)	0.874
	Internal validation cohort	0.885 (0.816–0.922)	0.75 (36/48)	1 (14/14)	0.806 (50/62)	1 (36/36)	0.538 (14/26)	0.857
	External validation cohort	0.851 (0.720–0.919)	0.8 (24/30)	0.846 (11/13)	0.814 (35/43)	0.923 (24/26)	0.647 (11/17)	0.857

PPV, positive predictive value; NPV, negative predictive value.

In the training cohort, the AUC of the combined model [AUC, 0.887 (95% CI: 0.827–0.940)] was significantly better than that of the radiomics model [AUC, 0.813 (95% CI: 0.743–0.877); p = 0.011] but not significantly different from that of the clinical factor model [AUC, 0.803 (95% CI: 0.723–0.876); p = 0.873]. The LRT indicated that there was a statistically significant improvement after the inclusion of Rad-score in the clinical factor model (p< 0.001) and after the inclusion of the Independent clinical predictors in the radiomics model (p = 0.036; **Table 4**). In the internal validation cohort, the combined model [AUC, 0.885 (95% CI: 0.816–0.922)] presented higher predictive efficacy than both the clinical factor model [AUC, 0.763 (95% CI: 0.603–0.841); p = 0.031] and the radiomics signature [AUC, 0.787 (95% CI: 0.606–0.825); p = 0.011]. In the external validation cohort, the combined model achieved an AUC of 0.851 (95% CI: 0.817–0.718) and showed similar predictive performance with the internal validation cohort.

**Table 4:** Comparison among the three models.

	Clinical model	Radiomics model	Combined model
Likelihood ratio	13.49	27.66	34.31
p value	<0.001*	0.036^#^	

*Comparison of the performance of the clinical model and the combined model.

^#^Comparison of the performance of the radiomics model and the combined model.

**Figure 6:**
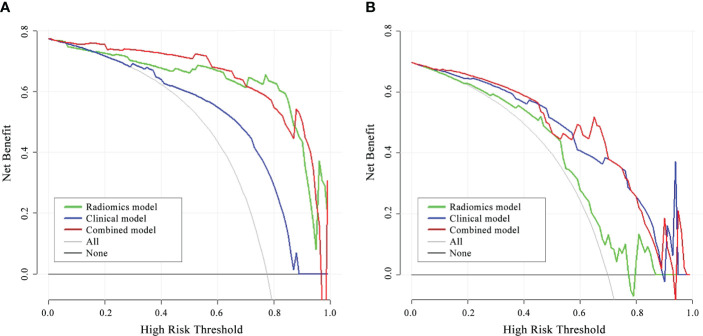
DCA of the three models in the internal validation cohort **(A)** and external validation cohort **(B)**. The red line, blue line, and green line stand for net benefit of the combined model, the clinical model, and the radiomics model, respectively. The gray line indicates the hypothesis that all patients were Group II CTD-ILD, while the black line is on behalf of the assumption that no patients were Group II CTD-ILD. The net benefit of the combined model is higher than that of the other two models and treating all or none of the patients across the majority range of reasonable threshold probabilities.

The DCA for the three models presented that the combined model performed better than the clinical model and the radiomics model in distinguishing between different stages of CTD-ILD across the majority of the range of reasonable threshold probabilities (**Figure 6**).

## Discussion

The present study showed that the combined model, which incorporated the CT-based Rad-score and clinical variables, had favorable predictive efficacy to distinguish different ILD-GAP stage patients with an AUC of 0.887, 0.885, and 0.851 in the training, internal validation, and external validation cohorts, respectively. In the present study, clinical variables and visual characteristics on CT image were enrolled. Multiple logistic regression analysis revealed that a larger number of cigarettes smoked and traction bronchiectasis on CT were independent predictors. Only 30 patients (14.85%) ever smoked in our dataset, and we believe it is because the number of male patients is smaller (n = 55, 27.23%). This revealed not only actual gender distribution of the CTDs but also the significant influence that smoking exerted on the mortality of CTD-ILD patients. A clinical factor model to classify ILD-GAP stages was then developed, incorporating pack-years of smoking and traction bronchiectasis on CT image, and achieved a high AUC of 0.803, 0.763, and 0.817 in the training, internal validation, and external validation cohorts, respectively. Honeycombing was proven not associated with GAP stages in our study that, however, with traction bronchiectasis were both independent risk factors for mortality in some research (38, 39). This was probably because of some biases caused by the imbalance between the groups. Goh et al. (40) established a staging system using the extent of disease with 20% demarcation for predicting mortality. However, parenchymal extent was not an independent predictor in the GAP index system by multivariable logistic regression analysis (p = 0.053) in our study. This was probably because Goh’s model built for SSc-ILD patients might not be applicable for all kinds of CTD-ILDs. Another reason might be that we did not find an optimal cutoff for parenchymal extent.

Radiomics based on CT image is an objective technique that provides a more reliable and comprehensive quantitative assessment of the images, not hindered by inter-reader variability. In the 1,409 radiomics features obtained from the CT images, eight higher-order texture features extracted from wavelet transformed images were acquired as remarkable elements to build the radiomics model, resulting in an AUC of 0.813, 0.787, and 0.718 in the training, internal validation, and external validation cohorts, respectively. Texture features can quantify information that is difficult to perceive visually, such as texture patterns or tissue distribution (41). Wavelet transform can level it up by obtaining multifrequency domain and multiscale image information after turning original images into different frequency domains (42, 43). For diseases that are difficult to be described by simple visual features, high-dimensional abstract feature extracted from wavelet transformed images can often provide different angles in capturing hidden information that is not easily observed by visual assessment.

Radiomics features have been proven to have potential for the severity estimation of CTD-ILD and treatment decision guidance (29). In recent years, rapidly developed radiomics provided large quantities of radiomics features, enabling full-scale characterization of the images beyond visual analysis. The clinical factor model comprising visual assessment performed significantly poorer in predicting GAP stage than the radiomics nomogram, indicating that information gathered from clinical and radiologic practice might be insufficient, and radiomics had the advantages of capturing and identifying the subtle features of ILD on CT images that were imperceptible to the radiologist but may imply prognosis. At present, there are limited studies focusing on applying radiomics in CTD-ILDs. Martini et al. (29) applied radiomics methods to develop a multivariable model and differentiate GAP stages in 60 patients with SSc, resulting in an AUC of 0.96. Instead of focusing on one single type of CTD, we expanded our samples up to 245 patients with different subtypes of CTD, which improved the universality of our radiomics nomogram. Most of the studies focused on predicting mortality of CTD-ILDs (27, 44); instead, we aimed to stage patients using baseline data and reduce potential unnecessary examinations. The promising results underlined the great potential of radiomics in ILDs. In the future, radiomics could be applied to support treatment decision. Previous studies have also proven that quantitative analysis can be applied to patients with ILDs. Kaya et al. (45) established a quantitative model with an AUC of 0.80 to predict GAP stages in 40 patients with idiopathic pulmonary fibrosis, proven to have the underlying possibility to outperform subjective visual inspection. Jacob et al. (46) proved that the volume of pulmonary blood vessels and surrounding fibrosis in the lungs independently predicted outcome in patients with RA-ILD. Radiomics methods provided much more information on the CT images that cannot be obtained by regular quantitative methods. In the present study, eight out of the nine features were high-order features, which may cover and exceed the quantitative features that previous studies have extracted.

Certain limitations of our study were as follows. First, cases in the two groups of our study were not balanced, therefore reflecting the prevalence of different GAP stages in our clinical population but may have an impact on our results. Second, there is still a gap for the assessment whether and which radiomics features were correlated with pathological manifestations in ILD. Thus, a multidisciplinary method combining clinical, radiological, and pathophysiological information may be proposed to guide individual-based treatment and benefit the prognosis. Third, there are certain holdbacks that radiomics could not be applied to all medical centers regarding technical limitations. The retrospective nature of this study may also hamper its reproducibility and generalization. Therefore, well-designed prospective radiomics trials as well as one-stop services that automatically segment images, extract features, and calculate the Rad-score need to be developed. Moreover, the result of this cross-sectional study may be less precise for using the verified ILD-GAP index system rather than actual mortality of the patients. The exact mortality risk and follow-up results will be investigated in our further research.

In conclusion, a CT-based radiomics nomogram was developed in our study. It revealed better efficacy in staging the severity of CTD-ILD on CT image than visual assessment, which implies that this noninvasive and quantitative method may impact the clinical decision-making process.

## Data availability statement

The original contributions presented in the study are included in the article/**Supplementary Material**. Further inquiries can be directed to the corresponding author.

## Ethics statement

The studies involving humans were approved by Shandong Provincial Hospital Affiliated to Shandong First Medical University. The studies were conducted in accordance with the local legislation and institutional requirements. The ethics committee/institutional review board waived the requirement of written informed consent for participation from the participants or the participants’ legal guardians/next of kin because this was a retrospective study.

## Author contributions

SQ, BK, and XW contributed to conception and design of the study. SQ, HL, HWL, BJ and HY organized the database. SQ performed the statistical analysis. SQ wrote the first draft of the manuscript. All authors contributed to manuscript revision, read, and approved the submitted version.
