# Supplementary material for: Non-contrast computed tomography-based radiomics for staging of connective tissue disease-associated interstitial lung disease
Source: Front Immunol. 2023 Oct 6;14:1213008. doi: 10.3389/fimmu.2023.1213008 (PMC10587549; doi:10.3389/fimmu.2023.1213008)
Supplement: Supplementary file 1 [file DataSheet_1.docx]

Supplementary Material

Non-contrast Computed Tomography-Based Radiomics for Staging of Connective Tissue Disease-associated Interstitial Lung Disease

Songnan Qin, Bingxuan Jiao, Bing Kang, Haiou Li, Hongwu Liu, Congshan Ji, Shifeng Yang, Hongtao Yuan, Ximing Wang*

*** Correspondence:** Ximing Wang: [wxming369@163.com](mailto:wxming369@163.com)

**Supplementary Table 1.** CT Image Acquisition Parameters

| **CT scanner** | **Center 1** | |  | **Center 2** |
| --- | --- | --- | --- | --- |
|  | 192-detector CT scanner (Force) | 64-detector CT scanner (Discovery 750 ) |  | 64-detector CT scanner (Discovery 750 ) |
| **Manufacturer** | Siemons | GE |  | GE |
| **Gantry rotation time (s)** | 0.5 | 0.5 |  | 0.5 |
| **Tube voltage (kV)** | 120 | 120 |  | 120 |
| **Tube current** | automatic tube current | automatic tube current |  | automatic tube current |
| **Convolution Kernel** | Br64//Bl64 | LUNG |  | LUNG |
| **Matrix** | 512×512 | 512×512 |  | 512×512 |
| **Increment (mm)** | 0.7 | 0.7 |  | 0.7 |

**Supplementary Table 2.** The distribution of CTD subtypes of the patients enrolled

| **CTD subtypes** | **Dataset 1** | |  | **Dataset 2** | |
| --- | --- | --- | --- | --- | --- |
|  | **Number of patients** | **Proportion** |  | **Number of patients** | **Proportion** |
| Systemic Lupus Erythematosus | 37 | 18.32% |  | 1 | 2.33% |
| Systemic Sclerosis | 27 | 13.37% |  | 2 | 4.65% |
| Rheumatoid Arthritis | 44 | 21.78% |  | 15 | 34.88% |
| Sjögren’s Syndrome | 17 | 8.42% |  | 3 | 6.98% |
| Idiopathic Inflammatory Myositis | 53 | 26.24% |  | 14 | 32.56% |
| Mixed Connective Tissue Disease | 24 | 11.88% |  | 8 | 18.60% |
| Sum | 202 | 100% |  | 43 | 100% |

**Supplementary Table 3.** Follow-up characteristics of the patients in dataset 1

| **Characteristics** | **All (n = 202)** | **Group I (n = 158)** | **Group II (n = 44)** |
| --- | --- | --- | --- |
| Gender |  |  |  |
| Male | 54 (26.7%) | 32 (20.3%) | 22 (50.0%) |
| Female | 148 (73.3%) | 126 (79.7%) | 22 (50.0%) |
| Median age (range), years | 56 (18-82) | 52 (18-79) | 64.5 (36-82) |
| Median follow-up time (range), months | 33 (2-89) | 35 (2-89) | 28 (10-87) |
| Died during follow-up | 16 (7.9%) | 7 (4.4%) | 9 (20.5%) |
| 3-year mortality (95% CI) | 9.0% (6.6-11.4%) | 5.3% (3.1-7.5%) | 22.5% (15.0-30.0%) |


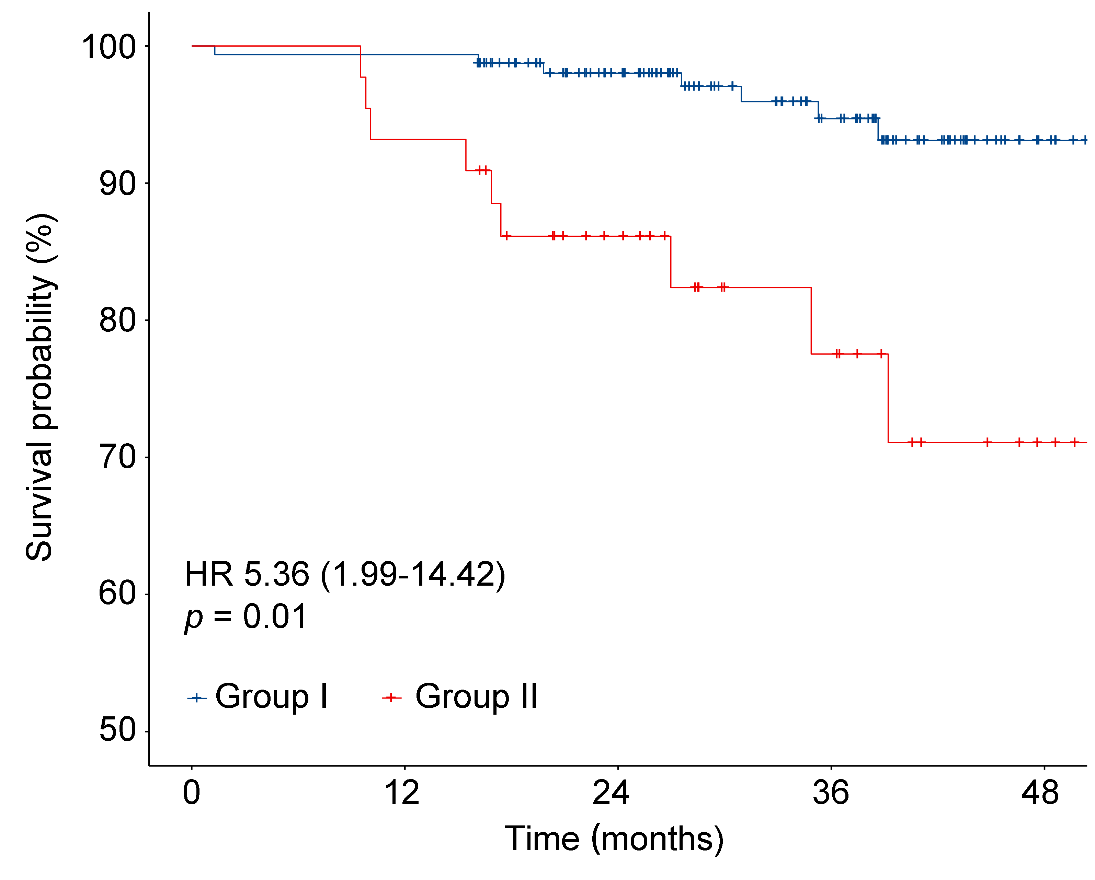


**Supplementary Figure 1.** Kaplan-Meier curves for overall survival (OS) defined as time to all-cause death in dataset 1

**Supplementary Table 4.** Independent risk factors in external-validation cohort

| **Variables** | **Univariable analysis** | |  | **Multivariable analysis** | |
| --- | --- | --- | --- | --- | --- |
|  | **Odds ratio (95% CI)** | ***p* value** |  | **Odds ratio (95% CI)** | ***p* value** |
| Pack-years of Smoking | 1.03 (1.01-1.06) | 0.011 |  | 1.04 (1.01-1.08) | 0.008 |
| Traction bronchiectasis | 6.40 (1.53-26.78) | 0.011 |  | 13.61 (2.05-90.32) | 0.007 |
| CI, confidence-interval | | | | | |

The features extracted can be divided into four main categories: (1) first-order statistical features such as mean, variance, maximum, median, and range describe the intensity information in the region of interest; (2) shape features such as volume, surface area, and maximum diameter reflect the shape and size of the region; (3) texture features may quantify regional differences in heterogeneity; and (4) higher-order statistical features included the first-order statistics and texture features derived from wavelet transformation such as exponential, square, square root, logarithm and wavelet (wavelet-LHL, wavelet-LHH, wavelet-HLL, wavelet-LLH, wavelet-HLH, wavelet-HHH, wavelet-HHL, wavelet-LLL) of the original images. Of the 1409 radiomics features extracted from CT images, 1367 were demonstrated to have a good inter- and intra-observer agreement (ICC: 0.7501- 1.0000). Seventy radiomics features showing significant differences between Stage I and Stage II CTD-ILD (P=0.001-0.050) by one-way ANOVA were enrolled into the LASSO logistic regression model. Finally, the radiomics signature was built using 9 features (Supplementary Table 2).

**Supplementary Table 5.** Selection results of radiomics features and coefficients for Rad-score building

| **Selected radiomics feature** | **Coefficient** |
| --- | --- |
| original_shape_Flatness | 0.05198 |
| wavelet-HHL_firstorder_Kurtosis | 0.01581 |
| wavelet-HLH_glcm_SumSquares | 0.03815 |
| wavelet-LHH_firstorder_Kurtosis | 0.04227 |
| wavelet-LHH_glcm_Autocorrelation | 0.02439 |
| wavelet-LHL_glrlm_GrayLevelVariance | -0.01671 |
| wavelet-LHL_glrlm_LowGrayLevelRunEmphasis | -0.00368 |
| wavelet-LHL_glszm_SizeZoneNonUniformityNormalized | -0.06562 |
| wavelet-LLL_glszm_SmallAreaEmphasis | 0.02710 |

**Supplementary Table 6.** The radiomics score of the training and validation sets

|  | **Training cohort (n=140)** | | |  | **Internal validation cohort (n=62)** | | |  | **External validation cohort (n=43)** | | |
| --- | --- | --- | --- | --- | --- | --- | --- | --- | --- | --- | --- |
|  | **Group I** | **Group II** | ***p*** |  | **Group I** | **Group II** | ***p*** |  | **Group I** | **Group II** | ***p*** |
| Rad-score |  |  | <0.001 |  |  |  | <0.001 |  |  |  | 0.033 |
| Median | 0.033 | -0.118 |  |  | 0.006 | -0.353 |  |  | -0.212 | -0.274 |  |
| Range | -0.343,  0.315 | -0.331,  0.233 |  |  | -0.489,  0.449 | -0.632,  0.008 |  |  | -0.473,  -0.074 | -0.724,  -0.132 |  |
